# Local field potentials in the auditory brain stem described by idealized biophysically-based models of the medial superior olive

**DOI:** 10.1186/1471-2202-13-S1-P61

**Published:** 2012-07-16

**Authors:** Joshua H Goldwyn, John Rinzel

**Affiliations:** 1 Center for Neural Science, New York University, New York, NY 10003, USA; 2 Courant Institute of Mathematical Sciences, New York University, New York, NY 10012, USA

## 

The medial superior olive (MSO) is one of the first stages in the mammalian auditory pathway to receive inputs from both ears. MSO neurons can act as exquisitely sensitive coincidence detectors and are able to detect interaural time differences with submillisecond precision [1]. Due to these specializations, the MSO is thought to play a critical role in spatial hearing [2].

A conspicuous feature of MSO electrophysiology is the presence of large, stimulus-evoked extracellular field potentials. These field potentials, known as the *auditory neurophonic*, have been observed since the earliest recordings from the auditory brain stem over half a century ago [3] and remain a topic of current interest [4]. The precise generators and functional consequences of the neurophonic remain largely unknown. We study these extracellular potentials in order to gain a new vantage point from which to advance both experimental and theoretical analyses of the MSO.

We begin with the assumption that postsynaptic current flow in MSO neurons is the dominant generator of the brain stem auditory neurophonic. We use a biophysically-based computational model of MSO neurons to simulate membrane currents [5]. To model extracellular currents, we make two idealizations. First, since MSO inputs are frequency-selective and strongly phase-locked to tonal stimuli, we assume synaptic inputs are synchronous. Second, since MSO neurons have a distinctive bipolar morphology with cell bodies that are approximately aligned in a two-dimensional sheet, we assume there is spatial symmetry in the distribution of synaptic currents. These assumptions of synchrony and symmetry lead to reduced models that we use to analyze the generation of the neurophonic.

We first show how monolateral excitatory input, when isolated on a single dendrite, creates an extracellular current sink near the site of excitation and a distributed current source along the remainder of the neuron. Simulated local field potentials share many qualitative features with published reports of the neurophonic. In particular, our model results are largely consistent with a classical description of the neurophonic as a dipole field generated by postsynaptic currents in MSO neurons [3,4]. The dipole theory makes no strong predictions about the dynamics of extracellular potentials, so we investigate how additional features of MSO activity – somatic inhibition, asymmetries between ipsilateral and contralateral inputs, subthreshold voltage-activated currents, bilateral inputs with varying delays – shape the evolution of the neurophonic. We conclude that an idealized model of MSO neurons with symmetric and synchronous inputs provides a useful tool for studying the neurophonic. Ongoing work seeks to relax these simplifying assumptions and to explore how local field potentials in the brain stem may influence the coincidence detection properties of MSO cells.
